# COVID-19 vaccine or booster hesitancy among children aged 6 month-5 years, 5–11 years, and 12–17 years in the United States: An analytic cross-sectional study

**DOI:** 10.1016/j.pmedr.2023.102436

**Published:** 2023-09-22

**Authors:** Chulwoo Park, Pyramida Vagoyan Zabala, Airi Irene Trisnadi

**Affiliations:** a Department of Public Health and Recreation, San José State University, San Jose, CA, United States; b Department of Psychology, San José State University, San Jose, CA, United States

**Keywords:** COVID-19 vaccine, COVID-19 booster, Vaccination hesitancy, Children, Household Pulse Survey, Cross-sectional study

## Abstract

With the increased accessibility of COVID-19 vaccine, many households have had concerns when vaccinating children, leading to vaccine hesitancy. This study examined the COVID-19 vaccine and booster hesitancy among children aged 6 months-5 years, 5–11 years, and 12–17 years in the United States. We analyzed data from Phase 3.8 (March 1, 2023 to May 8, 2023) of the Household Pulse Survey (HPS) collected by the U.S. Census Bureau. We conducted survey-weighted multiple logistic regression models in vaccine hesitancy among respondents with children from those three different age groups, controlling for various demographic factors such as COVID-19 vaccination status, COVID-19 positive test results, race/ethnicity, gender at birth, age, region, marital status, educational attainment, household income, health insurance, and children’s school type. The percentage of respondents indicating hesitancy towards vaccinating their children (expressing uncertainty, probably not, or definitely not) decreased as their children's age increased. Specifically, the proportion was 57.4% for children aged 6 months-5 years, 43.3% for children aged 5–11 years, and 25.9% for children aged 12–17 years. Concerns about possible side effects of the COVID-19 vaccine were the most prevalent among respondents who expressed vaccine hesitancy, regardless of the level of hesitancy, while those with strong hesitancy showed higher proportions of not believing their children need a vaccine, lack of trust in COVID-19 vaccines and the government, and parents/guardians not vaccinating their children. This study provide insight into our current situation, aiming to build assurance among households regarding the efficacy and benefits of COVID-19 vaccines for children of all ages.

## 1 Introduction

With the COVID-19 pandemic starting in late 2019 and reaching its peak in December of 2020, over 1.1 million people have lost their lives within the last three years (CDC, 2020). Among those reported in the U.S., approximately 1,000 of the deaths were of children between 5 and 18 years old and 800 deaths were under the age of 4 years old (CDC, 2023a). To prevent COVID-19, original (monovalent) vaccines, including the Pfizer-BioNTech, Moderna, Novavax, and J&J/Janssen, were released in the United States (CDC, 2023b). On December 11, 2020, the first Pfizer-BioTech vaccine was released for Emergency Use Authorizations (EUA) by the Food and Drug Administration (FDA) (CDC, 2023c). On May 10, 2021, FDA expanded the EUA of Pfizer-BioTech vaccine to include children ages 12–17 years old, for younger children ages 5–11 years on October 29, 2021, and for infants 6 months old to 5 years old on June 17, 2022 (CDC, 2023c). A few months later, CDC announced that the updated vaccines (bivalent) became available for all children between 12 and 17 years old on September 1, 2022, for those ages 6–11 years old on October 15, 2022, and children 6 months old to 5 years old became eligible starting December 9, 2022 (CDC, 2023b).

As the COVID-19 vaccinations became more accessible for the public including young children, controversies regarding the safety of vaccines presented a sense of hesitancy for individuals, especially for parents who showed concerns about vaccinating their children (Ruiz & Bell, 2022). Misconceptions and misinformation of the COVID-19 vaccines often times leads to parents having doubts of the vaccine efficacy (Garett and Young, 2021, Ullah et al., 2021, Skafle et al., 2022). Common reasons for vaccine hesitancy included distrust of vaccine safety, distrust in the government or pharmaceutical industry, beliefs that vaccines are unnecessary, and concerns that their children are too young (Fisher et al., 2022, Ruiz and Bell, 2022). These concerns were especially strong among parents with children under 5 years old (Fisher et al., 2022). Furthermore, several factors contributed to the differences in vaccine hesitancy such as the parents’ level of vaccine knowledge, belief in vaccine conspiracy, gender, family income, education, and political stance (Ruiz & Bell, 2022). Many planned to wait or were considering to receive the vaccine later after seeing that the vaccines are safe as they showed concerns of the side effects as well as concerns that COVID-19 vaccines were developed too quickly (Ngyuen et al., 2022b; Ruiz & Bell, 2022). Such vaccines hesitance has affected the vaccination status among young children. According to the trends of COVID-19 vaccine confidence by CDC, between April 30, 2023 and May 27, 2023, 88.2% of all adults over the age of 18 were vaccinated (CDC, 2020). On the other hand, for children 6 months old to 17 years old, only 40.2% have had at least one dose and 44.9% of those were “probably or definitely will not get vaccinated” (CDC, 2020).

Previous studies have focused on vaccine hesitancy towards children vaccination (Fisher et al., 2022, Lendon et al., 2021, Murthy et al., 2023, Nguyen et al., 2022a, Nguyen et al., 2022b). However, the relationship between vaccine or booster hesitancy across three children age groups has not been extensively researched. Therefore, the purpose of study was to examine the factors contributing to households’ vaccine or booster hesitancy for COVID-19 across three different age groups (6 months-5 years, 5–11 years, and 12–17 years) using the most recent Phase data (Phase 3.8) from the Household Pulse Survey (HPS) (United States Census Bureau, 2023).

## 2 Methods

The Household Pulse Survey (HPS) was established by the U.S. Census Bureau, the designated federal statistical agency, in collaboration with multiple federal agencies, to collect data on household experiences from adults aged 18 years and older on a biweekly basis during the COVID-19 pandemic across the United States. Under federal regulations for human subjects (45 CFR Part 46), this census data did not require IRB review because the data originated from publicly available sources and were deidentified, uncoded, and stripped of identifiers. The sampled housing units that HPS used was from the Census Bureau’s Master Address File (MAF). Households’ email and mobile telephone numbers from the Census Bureau Contact were added to MAF to distribute the survey through a rapid deployment internet response system. For this study, we conducted an analytic cross-sectional analysis using the most recent Phase as of July 2023 (Phase 3.8), collected from March 1, 2023 to May 8, 2023 (Week 55: March 1–March 13, 2023; Week 56: March 29–April 10, 2023; and Week 57: April 26–May 8, 2023). The total number of respondents during these selected three periods was 193,955, with response rates of 6.7% for Week 55, 5.7% for Week 56, and 5.5% for Week 57. Among them, we focused on households with children belonging to one of three age groups: 1) children aged 6 months-5 years (hereinafter children under 5), 2) children aged 5–11 years (hereinafter children 5–11), and 3) children aged 12–17 years (hereinafter children 12–17). Households with multiple age groups of children were excluded from the analysis. The final sample sizes for each age group were 11,736 for children aged 6 months to 5 years, 12,744 for children aged 5 to 11 years, and 18,620 for children aged 12 to 17 years.

In our analysis, we utilized 80 replicate weights derived from successive difference replications to calculate proportions and survey-weighted 95% confidence intervals (CIs). All percentages and confidence intervals (CI) in this study were weighted to represent the U.S. population. We conducted a demographic analysis, as presented in Table 1. Furthermore, we employed separate survey-weighted multiple logistic regression models for respondents with children under 5, children 5–11, and children 12–17 and estimated the adjusted odds ratio (aORs), as shown in Table 2, Table 4, Table 5. HPS asked about the likelihood of vaccinating children to those who have not yet vaccinated or received booster shots for their children, providing the following response options: 1) Definitely get the children a vaccine (hereinafter “definitely”), 2) Probably get the children a vaccine (hereinafter “probably”), 3) Be unsure about getting the children a vaccine (hereinafter “unsure”), 4) Probably NOT get the children a vaccine (hereinafter “probably NOT”), 5) Definitely NOT get the children a vaccine (hereinafter “definitely NOT”), and 6) I do not know the plans for vaccinating the children under 5 in my household.

**Table 1:** Demographic characteristics of households with children under 5, children 5–11, and children 12–17 in the United States, Phase 3.8 (March 1, 2023 to May 8, 2023).

Characteristics of respondents from households	Respondents with children < 5				Respondents with children 5–11				Respondents with children 12–17		
	Unweighted n	Weighted %	Weighted 95% CI		Unweighted n	Weighted %	Weighted 95% CI		Unweighted n	Weighted %	Weighted 95% CI
**Received the COVID-19 vaccine**	n = 11,688				n = 12,670				n = 18,478		
Yes	9,653	76.1	(73.2, 79.1)		10,516	77.1	(75.3, 78.8)		15,433	80.0	(78.3, 81.7)
No	2,035	23.9	(20.9, 2.8)		2,154	22.9	(21.2, 24.7)		3,045	20.0	(18.3, 21.7)
**Tested positive for COVID-19**	n = 11,387				n = 12,448				n = 18,245		
Yes	7,581	62.6	(59.6, 65.5)		8,018	60.3	(58.5, 62.1)		11,211	56.7	(53.3, 60.1)
No	3,806	37.4	(34.5, 40.4)		4,430	39.7	(37.9, 41.5)		7,034	43.3	(39.9, 46.7)
**Race/ethnicity**	n = 11,735				n = 12,743				n = 18,617		
Non-Hispanic White	8,228	57.1	(48.6, 65.7)		8,744	53.5	(48.9, 58.1)		12,777	52.7	(48.6, 56.9)
Non-Hispanic Black	911	12.0	(10.9, 13.1)		1,120	13.0	(9.3, 16.7)		1,711	12.5	(6.7, 18.2)
Non-Hispanic Asian	743	6.6	(4.7, 8.4)		776	6.4	(5.1, 7.7)		1,071	6.1	(4.2, 7.9)
Non-Hispanic Other Race	533	4.6	(3.9, 5.2)		619	4.7	(4.0, 5.4)		845	4.5	(4.0, 5.0)
Hispanic	1,320	19.7	(12.7, 26.7)		1,484	22.4	(16.0, 28.9)		2,267	24.2	(16.2, 32.2)
**Sex at birth**	n = 11,735				n = 12,743				n = 18,617		
Male	4,790	45.4	(43.8, 47.1)		5,023	46.8	(39.7, 53.8)		7,256	49.2	(41.6, 56.9)
Female	6,945	54.6	(52.9, 56.2)		7,720	53.2	(46.2, 60.3)		11,361	50.8	(43.1, 58.4)
**Age**	n = 11,735				n = 12,743				n = 18,617		
18–29	1,975	25.6	(21.2, 30.0)		531	11.0	(7.8, 14.2)		827	16.0	(3.0, 29.0)
30–39	6,421	45.3	(30.7, 59.8)		3,783	30.9	(18.8, 43.0)		1,555	9.3	(2.4, 16.3)
40–49	2,104	14.4	(11.8, 17.0)		5,880	34.3	(28.3, 40.2)		7,789	35.0	(28.5, 41.6)
50–59	645	7.3	(-0.0, 14.5)		1,567	11.6	(7.8, 15.5)		6,385	27.9	(26.7, 29.0)
60–69	430	5.3	(-0.9, 11.6)		720	8.8	(1.1, 16.5)		1,491	8.2	(7.5, 8.9)
70+	160	2.2	(-0.6, 5.0)		262	3.4	(0.0, 6.8)		570	3.6	(2.7, 4.4)
**Region**	n = 11,735				n = 12,743				n = 18,617		
Northeast	1,741	16.0	(11.9, 20.2)		1,986	17.7	(15.1, 20.4)		2,650	17.2	(15.3, 19.1)
South	3,821	40.3	(38.8, 41.7)		4,137	39.0	(35.7, 42.4)		5,980	38.3	(37.0, 39.6)
Midwest	2,556	20.7	(18.1, 23.3)		2,706	19.0	(13.3, 24.7)		4,159	20.1	(16.9, 23.3)
West	3,617	23.0	(17.2, 28.9)		3,914	24.2	(19.1, 29.3)		5,828	24.4	(18.8, 30.0)
**Marital Status**	n = 11,714				n = 12,713				n = 18,563		
Now Married	9,246	69.9	(68.3, 71.6)		8,566	60.5	(51.5, 69.4)		11,922	58.3	(45.1, 71.5)
Widowed/ Divorced/ Separated	901	9.7	(7.0, 12.4)		2,280	16.6	(8.6, 24.7)		4,273	17.9	(0.8, 35.1)
Never Married	1,567	20.4	(17.7, 23.0)		1,867	22.9	(21.2, 24.6)		2,368	23.8	(19.6, 27.9)
**Educational Attainment**	n = 11,735				n = 12,743				n = 18,617		
Less than or high school	228	6.8	(3.6, 10.1)		340	9.2	(7.0, 11.3)		629	11.0	(9.8, 12.1)
High school or equivalent	1,206	26.6	(21.9, 31.2)		1,580	31.4	(24.1, 38.7)		2,457	32.1	(28.7, 35.6)
Some college, but degree not received or is in progress	1,948	18.6	(17.5, 19.7)		2,594	19.5	(18.4, 20.6)		4,079	20.6	(18.3, 22.9)
Associate degree	1,011	9.5	(8.4, 10.5)		1,252	9.5	(8.0, 11.0)		2,110	8.6	(7.1, 10.2)
Bachelor’s degree	3,903	21.5	(17.2, 25.8)		3,590	16.4	(12.7, 20.1)		4,809	14.7	(13.0, 16.4)
Graduate degree	3,439	17.0	(13.0, 21.1)		3,387	14.0	(10.3, 17.8)		4,533	12.9	(10.8, 15.1)
**Household Income**	n = 9,150				n = 10,061				n = 14,669		
Less than $25,000	528	9.3	(8.1, 10.5)		769	10.8	(6.9, 14.7)		1,114	10.8	(4.6, 17.0)
$25,000 - $34,999	484	9.4	(7.9, 10.9)		624	9.4	(8.1, 10.7)		940	10.0	(8.7, 11.3)
$35,000 - $49,999	685	11.2	(9.0, 13.4)		866	11.6	(9.7, 13.5)		1,258	11.1	(9.5, 12.8)
$50,000 - $74,999	1,134	14.9	(13.7, 16.0)		1,363	16.5	(12.9, 20.1)		2,027	15.1	(13.2, 17.0)
$75,000 - $99,999	1,269	14.4	(13.1, 15.7)		1,253	13.6	(11.1, 16.1)		1,940	13.1	(11.1, 15.1)
$100,000 - $149,999	2,018	18.1	(16.7, 19.4)		2,058	16.7	(15.4, 18.0)		2,978	17.7	(14.9, 20.6)
$150,000 - $199,999	1,138	9.7	(8.8, 10.5)		1,263	9.6	(8.8, 10.4)		1,799	9.7	(7.4, 12.0)
$200,000 and above	1,894	13.1	(10.2, 16.0)		1,865	11.7	(9.9, 13.6)		2,613	12.5	(10.8, 14.2)
**Health Insurance**	n = 11,735				n = 12,743				n = 18,617		
Yes	9,281	70.8	(68.5, 73.1)		10,140	71.1	(68.7, 73.5)		14,868	70.1	(65.1, 75.1)
No	2,454	29.2	(26.9, 31.5)		2,603	28.9	(26.5, 31.3)		3,749	29.9	(24.9, 34.9)
**Children’s School Type**	n = 11,658				n = 12,727				n = 18,586		
Public	844	10.8	(8.5, 13.1)		10,120	80.6	(78.7, 82.6)		15,151	81.0	(78.9, 83.1)
Private	389	2.7	(2.0, 3.5)		1,312	8.8	(7.9, 9.6)		1,508	6.8	(6.1, 7.5)
**Homeschooled**	120	1.3	(0.5, 2.1)		360	2.7	(1.9, 3.5)		618	3.5	(2.2, 4.7)
None	10,073	83.0	(80.6, 85.4)		522	5.0	(4.0, 6.0)		480	4.2	(3.5, 5.0)
Combined	232	2.1	(1.6, 2.6)		413	2.9	(2.2, 3.6)		829	4.5	(3.7, 5.3)

NOTE: ▪ Health insurance (yes/no): yes for having either public or private insurance. ▪ In the school type, combined includes the combination of public and private, public and homeschooled, or private and homeschooled for those who with more than one child within the same age group.

**Table 2:** Proportion of vaccine or booster hesitancy for children under 5, children 5–11, and children 12–17 in the United States, Phase 3.8 (March 1, 2023 to May 8, 2023).

Characteristics of respondents from households	Respondents with children < 5 (n = 8,276)						Respondents with children 5–11 (n = 9,508)						Respondents with children 12–17 (n = 14,092)				
	Unweighted n	Weighted %	Weighted 95% CI	aOR	aOR 95% CI		Unweighted n	Weighted %	Weighted 95% CI	aOR	aOR 95% CI		Unweighted n	Weighted %	Weighted 95% CI	aOR	aOR 95% CI
**Overall**	5,124	57.4	(55.4, 59.3)	–	–		4,460	43.3	(42.0, 44.6)	–	–		4,229	25.9	(24.1, 27.7)	–	–
**Received the COVID-19 vaccine**																	
Yes (ref.)	3,480	47.1	(45.2, 48.9)	–	–		2,686	30.5	(29.0, 32.0)	–	–		1,868	12.7	(11.7, 13.6)	–	–
No	1,640	91.7	(88.9, 94.5)	11.39***	(6.66, 19.49)		1,774	88.7	(82.9, 94.5)	18.50***	(9.25, 37.00)		2,354	85.1	(82.7, 87.5)	30.35***	(21.04, 43.78)
**Tested positive for COVID-19**																	
Yes (ref.)	3,352	56.6	(53.6, 59.6)	–	–		2,838	42.6	(40.7, 44.5)	–	–		2,584	26.3	(23.7, 29.0)	–	–
No	1,630	56.9	(39.8, 46.5)	0.83*	(0.70, 0.97)		1,505	43.0	(40.3, 45.6)	0.84	(0.67, 1.06)		1,520	24.2	(22.0, 26.5)	0.90	(0.68, 1.19)
**Race/ethnicity**																	
Non-Hispanic White (ref.)	3,800	60.8	(58.9, 62.7)	–	–		3,241	47.8	(44.9, 50.8)	–	–		3,277	32.4	(30.8, 34.1)	–	–
Non-Hispanic Black	358	57.9	(50.6, 65.2)	1.00	(0.72, 1.41)		356	40.5	(31.7, 49.2)	0.53***	(0.40, 0.72)		308	21.7	(13.9, 29.5)	0.53***	(0.37, 0.77)
Non-Hispanic Asians	179	27.7	(21.9, 33.4)	0.49**	(0.30, 0.79)		104	17.9	(13.7, 22.2)	0.38***	(0.24, 0.60)		40	3.5	(2.4, 4.7)	0.12***	(0.06, 0.24)
Non-Hispanic Other Race	228	54.4	(48.3, 60.4)	0.87	(0.53, 1.42)		226	43.5	(37.2, 49.9)	0.74	(0.51, 1.07)		196	25.1	(19.9, 30.4)	0.55**	(0.35, 0.85)
Hispanic	559	58.2	(52.5, 64.0)	0.97	(0.68, 1.40)		533	41.7	(37.4, 46.0)	0.67*	(0.48, 0.95)		408	20.0	(14.3, 25.6)	0.55**	(0.37, 0.83)
**Gender at birth**																	
Male (ref.)	1,936	53.4	(49.7, 57.0)	–	–		1,584	40.6	(37.5, 43.8)	–	–		1,518	25.4	(23.4, 27.4)	–	–
Female	3,188	60.7	(58.5, 63.0)	1.34*	(1.04, 1.73)		2,876	45.7	(42.7, 48.6)	1.31*	(1.05, 1.62)		2,711	26.5	(23.3, 29.7)	0.94	(0.60, 1.47)
**Age**																	
18–29 (ref.)	1,188	72.2	(67.3, 77.2)	–	–		262	47.5	(29.2, 65.8)	–	–		132	22.6	(11.4, 33.8)	–	–
30–39	2,713	52.0	(47.6, 56.3)	0.76	(0.58, 1.00)		1,688	52.6	(49.7, 55.4)	2.17	(0.84, 5.57)		596	40.7	(35.3, 46.1)	1.89	(0.57, 6.34)
40–49	745	48.8	(40.6, 57.0)	0.64*	(0.42, 0.96)		1,677	35.3	(32.7, 37.9)	1.39	(0.49, 3.95)		1,963	29.7	(27.2, 32.1)	1.34	(0.44, 4.04)
50–59	270	62.8	(47.1, 78.5)	1.50	(0.65, 3.47)		516	42.1	(36.7, 47.5)	1.91	(0.56, 6.48)		1,153	19.5	(17.9, 21.2)	1.00	(0.36, 2.73)
60–69	148	50.9	(39.0, 62.7)	0.46*	(0.25, 0.86)		245	42.5	(34.0, 51.1)	1.51	(0.52, 4.36)		295	24.7	(18.5, 31.0)	1.50	(0.34, 6.59)
70+	60	59.3	(44.7, 73.9)	0.64	(0.30, 1.39)		72	34.4	(21.8, 47.1)	0.87	(0.22, 3.34)		90	19.2	(7.6, 30.8)	1.37	(0.56, 3.36)
**Region**																	
Northeast (ref.)	664	56.5	(51.7, 61.3)	–	–		506	33.0	(29.0, 37.0)	–	–		368	19.8	(15.5, 24.1)	–	–
South	1,768	61.6	(57.8, 65.3)	1.09	(0.70, 1.70)		1,652	51.4	(49.1, 53.7)	1.92***	(1.50, 2.46)		1,525	28.9	(26.9, 30.9)	1.04	(0.75, 1.44)
Midwest	1,210	58.1	(54.2, 62.1)	0.76	(0.46, 1.26)		1,040	45.8	(40.7, 50.9)	1.41*	(1.03, 1.93)		1,098	28.4	(21.1, 35.6)	0.98	(0.70, 1.38)
West	1,482	50.3	(46.4, 54.2)	0.75*	(0.57, 0.98)		1,262	36.4	(33.5, 39.3)	1.14	(0.86, 1.50)		1,238	23.6	(18.2, 29.1)	1.03	(0.43, 2.46)
**Marital Status**																	
Now Married (ref.)	3,920	53.6	(51.5, 55.7)	–	–		2,749	39.5	(36.9, 42.1)	–	–		2,580	24.6	(23.2, 25.9)	–	–
Widowed/ Divorced/ Separated	394	63.5	(53.2, 73.8)	0.88	(0.53, 1.46)		904	47.6	(44.2, 51.1)	1.23	(0.97, 1.56)		1,055	28.8	(26.5, 31.0)	0.94	(0.66, 1.34)
Never Married	805	69.2	(64.2, 74.1)	1.00	(0.73, 1.37)		800	50.8	(45.2, 56.5)	0.99	(0.63, 1.55)		583	27.1	(22.0, 32.2)	1.12	(0.82, 1.53)
**Educational Attainment**																	
Less than or high school (ref.)	92	63.8	(49.9, 77.6)	–	–		147	45.9	(38.2, 53.6)	–	–		161	22.8	(16.2, 29.4)	–	–
High school graduate or equivalent	685	70.2	(60.9, 79.5)	1.63	(0.40, 6.64)		789	53.6	(46.7, 60.4)	1.04	(0.50, 2.15)		800	34.5	(31.5, 37.5)	1.31	(0.57, 3.00)
Some college, but degree not received or is in progress	1,061	65.5	(61.2, 69.9)	1.34	(0.34, 5.25)		1,149	48.6	(44.0, 53.2)	1.15	(0.74, 1.80)		1,185	25.8	(17.7, 33.9)	0.98	(0.56, 1.72)
Associate degree	585	66.8	(59.3, 74.3)	1.57	(0.38, 6.40)		591	48.6	(42.7, 54.5)	1.26	(0.77, 2.08)		647	31.5	(28.2, 34.8)	1.26	(0.60, 2.62)
Bachelor’s degree	1,650	48.9	(46.4, 51.3)	0.97	(0.34, 2.78)		1,133	34.7	(31.9, 37.5)	0.74	(0.45, 1.23)		943	20.3	(18.1, 22.5)	1.07	(0.47, 2.45)
Graduate degree	1,051	35.6	(32.1, 39.1)	0.68	(0.20, 2.29)		651	21.0	(19.0, 23.0)	0.47**	(0.26, 0.83)		493	11.2	(9.8, 12.6)	0.72	(0.31, 1.69)
**Household Income**																	
Less than $25,000 (ref.)	277	70.7	(61.6, 79.8)	–	–		344	50.2	(42.0, 58.4)	–	–		351	33.3	(25.9, 40.6)	–	–
$25,000–34,999	266	68.3	(52.7, 83.8)	1.16	(0.38, 3.52)		280	46.3	(34.3, 58.4)	1.06	(0.69, 1.62)		261	28.8	(21.6, 35.9)	1.30	(0.33, 5.10)
$35,999–49,999	343	60.6	(42.7, 78.6)	0.84	(0.41, 1.72)		379	46.1	(40.7, 51.5)	1.15	(0.82, 1.61)		363	30.2	(24.8, 35.6)	0.93	(0.64, 1.36)
$50,000–74,999	593	60.9	(49.8, 72.1)	0.97	(0.53, 1.79)		562	49.2	(43.6, 54.8)	1.26	(0.87, 1.83)		584	30.5	(24.4, 36.6)	1.03	(0.72, 1.49)
$75,000–99,999	624	63.6	(59.1, 68.0)	1.23	(0.75, 2.01)		496	45.5	(40.8, 50.2)	1.20	(0.85, 1.69)		474	30.8	(26.3, 35.2)	1.03	(0.67, 1.58)
$100,000–149,999	890	53.9	(50.2, 57.6)	1.08	(0.66, 1.75)		661	37.4	(32.5, 42.3)	1.13	(0.73, 1.75)		635	23.0	(19.9, 26.1)	0.81	(0.53, 1.24)
$150,000–199,999	414	41.7	(37.0, 46.3)	0.75	(0.40, 1.39)		308	30.2	(22.9, 37.5)	0.90	(0.56, 1.44)		282	15.0	(12.0, 17.9)	0.55*	(0.32, 0.92)
$200,000 and above	498	30.5	(25.8, 35.2)	0.62	(0.37, 1.02)		337	21.3	(18.3, 24.3)	0.81	(0.53, 1.22)		246	10.4	(7.6, 13.1)	0.40***	(0.24, 0.64)
**Health Insurance**																	
Yes (ref.)	3,950	54.5	(52.5, 56.4)	–	–		3,318	39.9	(38.1, 41.7)	–	–		3,168	24.7	(23.1, 26.4)	–	–
No	1,174	65.4	(62.1, 68.7)	1.15	(0.65, 2.04)		1,142	52.4	(49.2, 55.6)	1.26	(0.68, 2.34)		1,061	29.0	(24.0, 34.1)	0.88	(0.52, 1.48)
**School Type**																	
Public (ref.)	381	55.3	(43.2, 67.5)	–	–		3,462	42.5	(40.9, 44.1)	–	–		3,392	25.1	(22.3, 27.9)	–	–
Private	179	52.7	(43.0, 62.4)	1.90	(0.52, 7.00)		457	44.2	(39.2, 49.2)	1.23	(0.89, 1.70)		271	25.4	(16.8, 34.1)	1.30	(0.78, 2.14)
Homeschooled	74	67.5	(51.7, 83.4)	2.12	(0.17, 27.27)		182	61.1	(49.5, 72.6)	1.65	(0.97, 2.81)		248	43.4	(35.3, 51.4)	1.15	(0.70, 1.89)
None	4,349	57.7	(55.9, 59.5)	1.57	(0.54, 4.55)		218	48.2	(39.5, 56.8)	1.48	(0.87, 2.51)		91	22.6	(14.7, 30.5)	0.56	(0.28, 1.11)
Combined	113	58.4	(48.0, 68.9)	2.05	(0.54, 7.79)		135	38.8	(30.1, 47.5)	1.00	(0.39, 2.56)		216	30.6	(23.1, 38.1)	1.14	(0.70, 1.85)

NOTE: ▪ Sample sizes in 3 groups (respondents with children under 5, children 5–11, and children 12–17) were, respectively. ▪ Dependent variable was a binary, hesitancy (unsure, probably NOT, or definitely NOT) vs. no hesitancy (already vaccinated, definitely, or probably). Adjusted odds ratio (aORs) were from a survey-weighted logistic regression model, controlling for demographic variables (received the COVID-19 vaccine, tested positive for COVID-19, gender at birth, age, region, marital status, educational attainment, household income, health insurance, and children’s school type). ▪ *P < 0.05 **P < 0.01 ***P < 0.001.

**Table 3:** Reasons for non-vaccination among households with vaccine or booster hesitancy (unsure, probably NOT, or definitely NOT) for children under 5, children 5–11, and children 12–17: a comparison between moderate hesitancy (unsure or probably NOT) and strong Hesitancy (definitely NOT) in the United States, Phase 3.8 (March 1, 2023 to May 8, 2023).

Reasons	Respondents with children < 5						Respondents with children 5–11						Respondents with children 12–17				
	Strong hesitancy (n = 2,744)		Moderate hesitancy (n = 2,380)		P-value		Strong hesitancy (n = 2,762)		Moderate hesitancy (n = 1,698)		P-value		Strong hesitancy (n = 3,147)		Moderate hesitancy (n = 1,082)		P-value
	Unweighted n	Weighted % (95% CI)	Unweighted n	Weighted % (95% CI)			Unweighted n	Weighted % (95% CI)	Unweighted n	Weighted % (95% CI)			Unweighted n	Weighted % (95% CI)	Unweighted n	Weighted % (95% CI)	
Concerns about possible side effects	1,611	52.2 (45.6, 58.7)	1,300	50.2 (45.4, 55.0)	**		1,555	50.9 (47.6, 54.2)	914	45.5 (37.3, 53.6)	0.107		1,673	50.0 (46.5, 53.5)	510	42.4 (30.4, 54.5)	***
Plan to wait and see if it is safe	411	12.9 (9.3, 16.5)	1,135	44.3 (39.0, 49.7)	***		253	8.7 (6.7, 10.8)	598	31.3 (26.5, 36.1)	***		205	6.3 (4.5, 8.1)	269	24.5 (19.8, 29.1)	***
Not sure if vaccine will work for children	223	6.9 (5.6, 8.2)	225	10.3 (6.4, 14.1)	0.095		215	7.3 (5.9, 8.7)	112	7.4 (5.1, 9.7)	0.142		196	5.7 (4.3, 7.2)	55	4.5 (2.9, 6.2)	0.167
Don’t believe children need a vaccine	1,270	39.1 (34.3, 44.0)	423	16.4 (14.0, 18.9)	***		1,185	37.7 (33.0, 42.4)	277	17.1 (11.7, 22.4)	***		1,149	32.5 (29.7, 35.3)	141	10.1 (7.5, 12.6)	***
Children not in high-risk group	1,078	32.6 (26.8, 38.4)	819	26.7 (17.3, 36.2)	***		993	30.6 (26.7, 34.5)	488	20.4 (16.4, 24.4)	***		984	26.2 (21.9, 30.4)	310	27.7 (21.4, 33.9)	0.107
Children’s doctor has not recommended it	490	15.9 (13.4, 18.3)	594	23.6 (20.5, 26.6)	***		349	11.4 (9.6, 13.3)	239	11.7 (9.2, 14.1)	0.168		279	7.8 (5.9, 9.7)	93	7.0 (4.9, 9.1)	0.787
Parents/ guardians do not vaccinate their children	174	6.3 (4.7, 7.9)	17	0.7 (0.3, 1.1)	***		172	7.0 (5.4, 8.6)	26	4.0 (-0.6, 8.6)	***		214	7.4 (5.9, 8.9)	29	3.9 (0.2, 7.6)	***
Don’t trust COVID-19 vaccines	1,479	53.3 (50.7, 55.8)	245	10.1 (5.9, 14.3)	***		1,511	55.8 (50.9, 60.8)	189	11.5 (9.5, 13.5)	***		1,832	59.0 (49.4, 68.6)	191	20.9 (14.1, 27.7)	***
Don’t trust the government	1,075	40.0 (36.0, 44.0)	189	8.8 (6.6, 11.0)	***		1,056	36.8 (34.1, 39.5)	128	9.0 (6.6, 11.4)	***		1,343	44.0 (35.6, 52.3)	120	14.1 (9.0, 19.1)	***
Other reasons	219	8.4 (5.1, 11.7)	188	7.7 (6.0, 9.4)	0.916		289	9.1 (6.6, 11.5)	148	9.5 (6.8, 12.1)	0.058		394	10.6 (6.4, 14.8)	137	11.7 (8.8, 14.7)	0.905

NOTE:

*P < 0.05 **P < 0.01 ***P < 0.001. ▪ Participants were able to choose more than one reason. ▪ Tests for proportion were conducted at the 0.05, 0.01, and 0.001 level of significance.

**Table 4:** Predictors of strong hesitancy (definitely NOT) among households with vaccine or booster hesitancy (unsure, probably NOT, or definitely NOT) for children under 5, children 5–11, and children 12–17 in the United States, Phase 3.8 (March 1, 2023 to May 8, 2023).

Characteristics of respondents (predictor)	Respondents with children < 5				Respondents with children 5–11				Respondents with children 12–17		
	aOR	95% CI	P-value		aOR	95% CI	P-value		aOR	95% CI	P-value
**Received the COVID-19 vaccine (Ref: Yes)**											
No	6.55	(4.65, 9.24)	***		5.46	(3.97, 7.52)	***		6.01	(4.30, 8.40)	***
**Tested positive for COVID-19 (Ref: Yes)**											
No	1.03	(0.73, 1.45)	0.870		1.03	(0.77, 1.38)	0.842		0.88	(0.54, 1.42)	0.597
**Race/ethnicity (Ref: Non-Hispanic White)**											
Non-Hispanic Black	0.52	(0.33, 0.81)	**		0.55	(0.32, 0.93)	*		0.40	(0.21, 0.76)	**
Non-Hispanic Asian	0.39	(0.18, 0.84)	*		0.48	(0.19, 1.25)	0.134		0.52	(0.15, 1.81)	0.307
Non-Hispanic Other Race	0.81	(0.34, 1.97)	0.650		1.23	(0.56, 2.69)	0.605		0.81	(0.37, 1.79)	0.600
Hispanic	0.69	(0.45, 1.06)	0.094		0.68	(0.46, 1.00)	*		0.66	(0.37, 1.18)	0.163
**Gender at birth (Ref: Male)**											
Female	0.88	(0.68, 1.14)	0.346		0.82	(0.61, 1.11)	0.199		0.87	(0.60, 1.25)	0.450
**Age (Ref: 18–29)**											
30–39	1.21	(0.89, 1.64)	0.227		2.06	(0.95, 4.44)	0.066		2.84	(1.01, 7.98)	*
40–49	1.63	(1.04, 2.55)	*		1.75	(0.86, 3.57)	0.121		2.48	(0.87, 7.04)	0.087
50–59	0.89	(0.55, 1.45)	0.636		1.66	(0.65, 4.22)	0.289		1.56	(0.62, 3.90)	0.343
60–69	1.09	(0.42, 2.82)	0.859		1.43	(0.53, 3.88)	0.477		2.83	(0.78, 10.31)	0.115
70+	0.70	(0.14, 3.59)	0.666		0.87	(0.14, 5.52)	0.880		2.72	(0.46, 16.25)	0.272
**Region (Ref: Northeast)**											
South	0.95	(0.58, 1.55)	0.845		0.81	(0.47, 1.40)	0.458		0.90	(0.47, 1.72)	0.760
Midwest	0.74	(0.49, 1.12)	0.159		0.89	(0.53, 1.48)	0.644		1.17	(0.57, 2.38)	0.671
West	0.71	(0.49, 1.02)	0.061		0.81	(0.49, 1.33)	0.394		0.96	(0.52, 1.79)	0.909
**Marital Status (Ref: Married)**											
Widowed/Divorced/ Separated	2.34	(0.78, 7.03)	0.130		1.34	(0.93, 1.94)	0.113		0.82	(0.57, 1.17)	0.274
Never Married	0.77	(0.52, 1.15)	0.204		1.11	(0.65, 1.89)	0.694		0.70	(0.40, 1.21)	0.204
**Educational Attainment (Ref: Less than or high school)**											
High school graduate or equivalent	1.26	(0.39, 4.06)	0.694		1.15	(0.55, 2.41)	0.711		0.98	(0.45, 2.14)	0.963
Some college, but degree not received or is in progress	0.95	(0.30, 2.99)	0.926		1.37	(0.73, 2.59)	0.329		1.20	(0.59, 2.44)	0.624
Associate’s degree	1.13	(0.35, 3.58)	0.840		1.11	(0.53, 2.37)	0.777		2.01	(1.01, 3.99)	*
Bachelor’s degree	0.76	(0.25, 2.33)	0.630		1.08	(0.61, 1.91)	0.792		1.98	(1.12, 3.51)	*
Graduate degree	0.53	(0.18, 1.61)	0.263		1.09	(0.56, 2.10)	0.805		2.08	(1.07, 4.06)	*
**Household Income (Ref: Less than $25,000)**											
$25,000 - $34,999	1.11	(0.56, 2.21)	0.757		0.78	(0.19, 3.19)	0.732		0.61	(0.14, 2.68)	0.512
$35,999 - $49,999	1.12	(0.52, 2.45)	0.768		0.76	(0.37, 1.56)	0.458		0.77	(0.26, 2.26)	0.631
$50,000 - $74,999	1.28	(0.71, 2.31)	0.416		1.05	(0.57, 1.91)	0.880		0.66	(0.26, 1.70)	0.386
$75,000 - $99,999	1.56	(0.87, 2.80)	0.135		1.58	(0.84, 2.97)	0.158		0.81	(0.33, 2.01)	0.653
$100,000 - $149,999	1.43	(0.76, 2.71)	0.272		1.35	(0.71, 2.58)	0.360		0.85	(0.34, 2.15)	0.731
$150,000 - $199,999	1.79	(0.87, 3.69)	0.113		1.52	(0.63, 3.65)	0.349		0.91	(0.31, 2.63)	0.859
$200,000 and above	1.19	(0.58, 2.46)	0.639		1.90	(0.88, 4.09)	0.102		1.49	(0.62, 3.57)	0.367
**Health Insurance (Ref: Public)**											
Private	1.71	(0.87, 3.36)	0.120		0.91	(0.46, 1.81)	0.786		0.94	(0.45, 2.00)	0.881
**School Type (Ref: Public)**											
Private	1.97	(0.78, 4.97)	0.149		1.37	(0.71, 2.66)	0.352		1.41	(0.73, 2.72)	0.307
Homeschooled	1.23	(0.24, 6.17)	0.803		1.19	(0.30, 4.77)	0.806		3.17	(1.12, 9.00)	*
None	1.78	(1.05, 3.01)	*		0.58	(0.35, 0.97)	*		1.08	(0.32, 3.66)	0.902
Combined	1.72	(0.72, 4.09)	0.219		1.06	(0.52, 2.17)	0.874		0.71	(0.32, 1.55)	0.386

NOTE:

*P < 0.05 **P < 0.01 ***P < 0.001. ▪ Dependent variable was a binary, strong hesitancy (definitely NOT) vs. moderate hesitancy (unsure or probably NOT). Adjusted odds ratio (aORs) were from a survey-weighted logistic regression model, controlling for demographic variables (received the COVID-19 vaccine, tested positive for COVID-19, gender at birth, age, region, marital status, educational attainment, household income, health insurance, and school type). ▪ Sample sizes for respondents with children under 5, children 5–11, and children 12–17 in survey-weighted logistic regression models were restricted to those who with vaccine hesitancy (Unsure, Probably NOT, or definitely NOT), 3,877, 3,357, and 3,175, respectively.

**Table 5:** Reasons for strong hesitancy (definitely NOT) among households with vaccine or booster hesitancy (unsure, probably NOT, or definitely NOT) for children under 5, children 5–11, and children 12–17 in the United States, Phase 3.8 (March 1, 2023 to May 8, 2023).

Reasons	Respondents with children < 5				Respondents with children 5–11				Respondents with children 12–17		
	aOR	95% CI	P-value		aOR	95% CI	P-value		aOR	95% CI	P-value
Concern about possible side effects	1.27	(0.89, 1.81)	0.180		1.27	(0.87, 1.85)	0.209		1.28	(0.80, 2.04)	0.304
Plan to wait and see if it is safe	0.21	(0.16, 0.27)	***		0.18	(0.12, 0.25)	***		0.22	(0.13, 0.39)	***
Not sure if vaccine will work on children	0.91	(0.58, 1.43)	0.688		1.10	(0.59, 2.07)	0.761		1.65	(0.70, 3.89)	0.254
Don’t believe children need a vaccine	3.46	(2.51, 4.78)	***		2.35	(1.27, 4.35)	**		4.39	(2.87, 6.70)	***
Children is not in high-risk groups	1.21	(0.93, 1.57)	0.157		1.45	(1.09, 1.92)	*		0.65	(0.48, 0.88)	**
Children’s doctor has not recommended it	0.67	(0.51, 0.87)	**		0.82	(0.57, 1.18)	0.284		1.66	(0.76, 3.60)	0.202
Parents/ guardians do not vaccinate their children	4.62	(1.46, 14.61)	**		2.52	(1.13, 5.62)	*		2.30	(0.55, 9.53)	0.251
Don’t trust COVID-19 vaccines	7.23	(5.50, 9.50)	***		8.23	(5.72, 11.85)	***		4.67	(2.54, 8.57)	***
Don’t trust the government	5.94	(4.02, 8.77)	***		5.53	(3.73, 8.20)	***		4.41	(2.40, 8.09)	***
Other reasons	1.00	(0.53, 1.90)	0.997		1.28	(0.73, 2.26)	0.394		0.92	(0.57, 1.48)	0.734

NOTE:

*P < 0.05 **P < 0.01 ***P < 0.001. ▪ Participants were able to choose more than one reason. ▪ Dependent variable was a binary, strong hesitancy (definitely NOT) vs. moderate hesitancy (Unsure or Probably NOT). Adjusted odds ratio (aORs) were from a survey-weighted logistic regression model, controlling for demographic variables (received the COVID-19 vaccine, tested positive for COVID-19, gender at birth, age, region, marital status, educational attainment, household income, health insurance, and children’s school type). ▪ Sample sizes for respondents with children under 5, children 5–11, and children 12–17 in survey-weighted logistic regression models were restricted to those who with vaccine hesitancy (Unsure, Probably NOT, or definitely NOT), 3,877, 3,357, and 3,175, respectively.

We used a binary dependent variable: vaccine hesitancy (unsure, probably NOT, or definitely NOT) versus no hesitancy (already vaccinated, definitely, or probably) for Table 2. For odds ratios, we used the first option within each of the variables present in the dataset (e.g., “yes,” “male, “18–29,” and “Northeast”) as the reference group. Regarding race/ethnicity, we combined two original variables, Hispanic Origin (RHISPANIC) and Race (RRACE), into a single variable. “White, Alone” was the first category in the RRACE variable, and we listed this nominal variable after combining it with the RHISPANIC variable in the following order: 1) non-Hispanic White (reference), 2) non-Hispanic Black, 3) non-Hispanic Asian, 4) non-Hispanic other race, and 5) Hispanic.

When comparing strong hesitancy with moderate hesitancy, the dependent variable was vaccine or booster rejection: strong hesitancy for children (definitely NOT) versus moderate hesitancy for children (unsure or probably NOT), as indicated in Table 4, Table 5. To determine the statistical significance of the comparisons between moderate hesitancy and strong hesitancy for each reason for not vaccinating children, we utilized tests for proportions at significance levels of 0.05, 0.01, and 0.001 (Table 3). All analyses were conducted using Stata/MP (version 14.2; StataCorp).

## 3 Results

### 3.1 Demographic information

We categorized the respondents into three groups based on their children’s age: 1) respondents with children under 5, 2) respondents with children aged 5–11, and 3) respondents with children aged 12–17. Respondents who had children falling into multiple age groups were excluded from the analysis. Overall, these three groups displayed similar demographic characteristics, as shown in Table 1. Across all three groups, the majority of respondents (76.1–80%) had received the COVID-19 vaccine. Additionally, more than half of the respondents (56.7–62.6%) either tested positive for COVID-19 or were informed by a doctor or healthcare provider that they had contracted the virus. The largest age group for respondents with children under 5, 5–11, and 12–17 were 30–39 (45.3%), 40–49 (34.3%), and 40–49 (35%) respectively. Respondents from the southern region (38.3–40.3%) and were currently married (58.3–69.9%) were the most prevalent across all groups. Furthermore, more than 70% of respondents had either public or private health insurance. In terms of schooling, a significant majority of children aged 5–11 (80.6%) and children aged 12–17 (81%) were attending public schools, while children under 5 (83%) were not enrolled in schools.

### 3.2 Proportion of vaccine or booster hesitancy

Overall, the proportion of respondents expressing vaccine or booster hesitancy for their children (unsure, probably NOT, or definitely NOT) decreased as their children’s age increased (57.4% [95% CI: 55.4–59.3] for children under 5, 43.3% [42.0–44.6] for children 5–11, and 25.9% [24.1–27.7] for children 12–17) (Table 2). Across all three age groups, respondents who had not received the COVID-19 vaccine showed a strong association with vaccine or booster hesitancy for their children (aORs: 11.39 [6.66–19.49], 18.50 [9.25–37.00], and 30.35 [21.04–43.78], respectively) at α = 0.001. Non-Hispanic Asians, in terms of race/ethnicity, were the least likely to demonstrate vaccine or booster hesitancy compared to non-Hispanic Whites across all three age groups. Moreover, as children’s age group increased, the protective factor against vaccine or booster hesitancy also increased among non-Hispanic Asians (aORs: 0.49 [0.30–0.79, *P* < 0.01], 0.38 [0.24–0.60, *P* < 0.001], and 0.12 [0.06–0.24, *P* < 0.001], respectively). Females showed a higher likelihood of vaccine or booster hesitancy than males for children under 5 (aOR: 1.34 [95% CI: 1.04–1.73]) and children 5–11 (aOR: 1.31 [1.05–1.62]) at α = 0.05. Additionally, for respondents with children 12–17, those earning $200,000 and above demonstrated a reduced likelihood of vaccine or booster hesitancy compared to those earning less than $25,000 (aOR: 0.40 [0.24–0.64], *P* < 0.001).

### 3.3 Reasons for not vaccinating their children

We calculated the proportion of the reasons for not getting children (under 5, 5–11, and 12–17) vaccinated among respondents who have neither vaccinated nor definitely would get their children a vaccine, stratified by vaccine or booster hesitancy into two groups, moderate hesitancy (unsure or probably NOT) and strong hesitancy (definitely NOT) (Table 3). Among the listed reasons, concerns about possible side effects of the COVID-19 vaccine were the most prevalent, ranging from 42.4% to 52.2%, regardless of the level of vaccine or booster hesitancy across all groups. Across all three age groups of children, a significantly lower proportion of respondents with strong hesitancy expressed the intention to wait and see if the vaccine is safe, compared to respondents with moderate hesitancy (12.9% vs. 44.3%, 8.7% vs. 31.3%, and 6.3% vs. 24.5%, respectively) at α = 0.001. In addition, compared to those with moderate hesitancy, respondents with strong hesitancy demonstrated statistically significantly higher proportions of not believing their children need a vaccine (39.1% vs. 16.4%, 37.7% vs. 17.1%, and 32.5% vs. 10.1%, respectively), parents/guardians not vaccinating their children (6.3% vs. 0.7%, 7.0% vs. 4.0%, and 7.4% vs. 3.9%, respectively), lacking trust in COVID-19 vaccines (53.3% vs. 10.1%, 55.8% vs. 11.5%, and 59.0% vs. 20.9%, respectively), and lacking trust in the government (40.1% vs. 8.8%, 36.8% vs. 9.0%, and 44.0% vs. 14.1%, respectively) at α = 0.001. Respondents with strong vaccine hesitancy towards children under 5 and children 5–11 showed significantly higher percentages of not believing that children are in high-risk group for COVID-19, as compared to respondents with moderate hesitancy (32.6% vs. 26.7% for children under 5, and 30.6% vs. 20.4% for children 5–11) at α = 0.001, while no significant difference was observed between strong hesitancy and moderate hesitancy for children 12–17 (26.2% vs. 27.7%).

### 3.4 Strong hesitancy among participants with vaccine or booster hesitancy

Among households with vaccine or booster hesitancy (unsure, probably NOT, or definitely NOT), respondents who did not receive the COVID-19 vaccine showed significant high predictor of strong hesitancy (definitely NOT) compared to those who received the COVID-19 vaccine (aORs: 6.55 [4.65–9.24], 5.46 [3.97–7.52], and 6.01 [4.30–8.40], respectively) at α = 0.001 across all children age groups (Table 4). Non-Hispanic Black were protective against strong hesitancy compared to non-Hispanic White across children under 5, children 5–11, and children 12–17 (aORs: 0.52 [0.33–0.81, *P* < 0.01], 0.55 [0.32–0.93, *P* < 0.05], and 0.40 [0.21–0.76, *P* < 0.01], respectively). Notably, respondents who attained an associate’s degree or higher education level and had children 12–17 demonstrated a significantly higher likelihood of strong hesitancy, as compared to respondents with less than a high school education level and children 12–17 (α = 0.05).

### 3.5 Reasons for not vaccinating their children among participants with vaccine or booster hesitancy

Aligned with the findings presented in Table 3, Table 5 shows aORs for reasons behind not vaccinating children. The comparison is made between the binary outcome variables of strong hesitancy (definitely NOT) and moderate hesitancy (unsure or probably NOT), presented in the order of children under 5, children 5–11, and children 12–17. The results indicate that the intention to wait and see if the vaccine is safe acted as a protective factor against strong hesitancy across all three children groups (aORs: 0.21 [0.16–0.27], 0.18 [0.12–0.25], and 0.22 [0.13–0.39], respectively) at α = 0.001. However, across all age groups of children, the reasons for not trusting COVID-19 vaccines (aOR: 7.23 [5.50–9.50], 8.23 [5.72–11.85], and 4.67 [2.54–8.57], respectively) and not trusting the government (aORs: 5.94 [4.02–8.77], 5.33 [3.73–8.20], and 4.41 [2.40–8.09], respectively) emerged as predictors of strong hesitancy (α = 0.001). Similarly, respondents with strong hesitancy were more likely to not believe that children would need a vaccine, compared to those with moderate hesitancy (aORs: 3.46 [2.51–4.78, *P* < 0.001], 2.35 [1.27–4.35, *P* < 0.01], and 4.39 [2.87–6.70, *P* < 0.001], respectively).

## 4 Discussion

This study provided insights into the current situation of vaccine or booster hesitancy for children under 18 years old by analyzing up-to-date information from HPS, enabling medical organizations or industries to reflect on the support systems available to the community. By considering the reasons for vaccine or booster hesitancy, the community can help households better understand and gain assurance about the benefits of vaccinations when making decisions regarding the vaccination of their children. Data on vaccine hesitancy among child populations have primarily relied on parental opinions (Fazel et al., 2021). Likewise, Gray and Fisher (2022) reported that COVID-19 vaccine uptake among children is contingent on their parents’ perception of the vaccine. However, a few studies delved into COVID-19 vaccine hesitancy among children and adolescents showing that they have contributed to decision-making around vaccines. Wang et al. (2022) emphasized that adolescent vaccine hesitancy presents a significant challenge in the global effort to combat the COVID-19 pandemic. Apart from parental opinions, Fazel et al. (2021) conducted a large, school-based self-report survey assessing the willingness of COVID-19 vaccination among students aged 9–18 years in England. Their findings indicated that 49.9% of students fell into the vaccine-hesitant category, with 37% being undecided and 12.9% opting out. Willis et al. (2021) reported the similar result, with more than half of youth aged 12–15 (58%) indicating some degree of COVID-19 vaccine hesitancy. Overall, both parental and children’s intentions regarding vaccination showed a similar pattern. Our findings indicate that the percentage of parents or guardians expressing hesitancy towards vaccinating their children decreased as the age of their children increased, aligning with the fact that older students aged 13–19 demonstrated a significantly stronger intention to get vaccinated compared to the younger age group (Scharff et al., 2022).

Between March 1, 2023 and May 8, 2023, 76.1–80% of respondents with children under 5, 5–11, and 12–17 reported to have received the COVID-19 vaccine and 56.7–62.6% have either tested positive for COVID-19 or were informed by a doctor or healthcare provider to have contracted the virus (Table 1). Ngyuen et al. (2022b) reported that respondents’ COVID-19 vaccine uptake was positively associated with the likelihood for getting their children vaccinated. In accordance with this finding, our study found that respondents who have not received the COVID-19 vaccine showed hesitancy to get children vaccinated or boosted (91.7% for children under 5, 88.7% for children 5–11, and 85.1% for children 12–17) (Table 2). In addition, we observed a decrease in COVID-19 vaccine hesitancy as the children’s age increased (Table 2), aligning with the findings from Ngyuen et al. (2022b) that the prevalence of COVID-19 vaccination was significantly higher among children 12–17 than children 5–11. Children 12–17 may be more capable of educating themselves on the safety and efficacy of the vaccine and conversing with their parents about getting vaccinated against COVID-19.

Non-Hispanic Asian respondents were found to have the lowest COVID-19 vaccination hesitancy for children in their households across all races and ethnicities. This finding aligns with the highest COVID-19 vaccination coverage among non-Hispanic Asians, as reported by Valier et al. (2023) and Na et al. (2023), who used different national data from the National Immunization Survey–Child COVID Module (NIS-CCM) and a national longitudinal survey, respectively. This could be related to the highest education and household income of Asian respondents (Na et al., 2023). In addition, female respondents with children under 5 and children 5–11 were more likely to have higher rates of hesitancy to vaccinate their children for COVID-19 compared to male respondents, aligning with recent studies (Beleche et al., 2021, Lendon et al., 2021, Zintel et al., 2022 Morales et al., 2022, Santibanez et al., 2023, Toshkov, 2023). The reasons why women were more likely to have COVID-19 vaccination hesitancy included questioning the safety of COVID-19 vaccines due to the rapid development with unknown long-term side effects and not believing that a vaccine is the only way to stop the pandemic, as well as not weighing the benefits of vaccines against possible risks (Toshkov, 2023).

When it comes to respondents’ educational level, we assumed that respondents with higher levels of education would be more inclined to vaccinate their children. However, our analysis showed that there was no statistically significant difference in vaccine hesitancy between respondents with children under 5 and children 5–11, regardless of their educational attainment. Interestingly, we observed a noteworthy trend among respondents with children 12–17. Those who had attained an associate’s degree, bachelor’s degree, or graduate degree showed a significantly higher likelihood of strong hesitancy compared to respondents with an education level below high school when it came to vaccinating their children 12–17. This outcome contradicts the conclusions drawn in other studies, where an educational level below a bachelor’s degree was associated with hesitancy towards routine childhood vaccinations and annual influenza vaccines (Kempe et al., 2020), as well as a lower parental educational level being linked to increased COVID-19 vaccine hesitancy (Scharff et al., 2022). This discrepancy may stem from differences in how education levels are categorized. In our study, we employed a detailed ordinal variable with seven categories, which included: 1) less than a high school diploma, 2) high school graduate or equivalent, 3) some college with no degree or degree in progress, 4) associate’s degree, 5) bachelor’s degree, and 6) graduate degree. In contrast, Scharff et al. (2022) compared the number of the college-educated adults in the household (i.e., none vs. one, none vs. two, and one vs. two), and Kempe et al. (2020) used a simpler variable for respondent education (i.e., high school or less, some college, and bachelor’s degree or higher).

In line with prior studies, among those who showed strong hesitancy, common reasons for not vaccinating their children were associated with perceived lack of necessity and mistrust towards the COVID-19 vaccines and government (Lendon et al., 2021, Singh et al., 2022, Wu and Zhang, 2022, Santibanez et al., 2023). Regardless of the level of vaccine or booster hesitancy (moderate or strong) among respondents who have neither vaccinated children nor definitely would get their children vaccinated, our study identified the prominent reason for hesitation towards vaccinating children for COVID-19 was concerns about safety, such as possible side effects. Across all age groups of children, respondents who reported a “wait and see” approach regarding the safety of the COVID-19 vaccine demonstrated a sense of caution rather than outright rejection of vaccination for children in their households. This cautious stance indicates that these individuals are monitoring the vaccine’s safety and efficacy, and they may be open to vaccinating children once they feel more confident about its benefits and potential risks. They might prioritize seeking assurance in the long-term safety profile of the vaccine before vaccinating their children. By intending to vaccinate children eventually, they demonstrate a willingness to protect their children and contribute to collective efforts in curbing the pandemic. This nuanced attitude towards vaccination highlights the importance of providing transparent and accurate information to address concerns and encourage informed decision-making among caregivers.

## 5 Limitations

The findings in this report are subject to at least six limitations. First, the findings of the study cannot be generalized to the general population due to the low response rates of Phase 3.8 of HPS (United States Census Bureau, 2023). Even though the survey weights might mitigate some of the bias between the true value and sample estimates, bias in the estimates may persist. Second, our study excluded respondents who have children in the household belonging to more than one age group which further limits the generalizability of the findings. Third, there may be recall bias in the reporting of children vaccination status by the selected respondent of the household. In addition, the selected respondents may not always be the parent/guardian of the children in the household and information reported on the children’s vaccination status may potentially be inaccurate. Fourth, information was self-reported by the respondents and social desirability bias may have been introduced. Fifth, information on socio-behavioral factors aside from reasons for not intending to vaccinate children were not collected in the HPS. Lastly, this study was designed as a cross-sectional study, which inherently imposes limitations on establishing causal relationships.

## 6 Conclusions

General public health efforts targeting the U.S. adult population may have an impact on vaccination rates among children, considering our findings that respondents’ vaccination status significantly predicts strong hesitancy toward vaccinating their children. Respondents’ vaccine or booster hesitancy was the most prevalent among those with the youngest age children (under 5). Therefore, public health initiatives aimed at increasing COVID-19 vaccination rates among children should be tailored to address the concerns about safety and mistrust expressed by parents of children within this age group. Effective strategies for disseminating accurate information about COVID-19 vaccines can encompass various approaches, including public health campaigns that utilize multiple media channels to provide information on vaccine safety and efficacy, school-based interventions engaging students and families through workshops and educational materials, and direct engagement by healthcare professionals such as doctors, nurses, and pharmacists with patients and their families. Additionally, community outreach and the use of mass and social media campaigns can help reach underserved populations. In this collective effort, ongoing research and collaboration between healthcare providers, public health experts, and the community will play an instrumental role in addressing vaccine hesitancy and ensuring the successful vaccination of children against COVID-19.

## Funding

This study was supported by the College of Health and Human Sciences, San José State University.

## Declaration of Competing Interest

The authors declare that they have no known competing financial interests or personal relationships that could have appeared to influence the work reported in this paper.

## Data Availability

Data will be made available on request.
